# Natural mega disturbances drive spatial and temporal changes in diversity and genetic structure on the toadfish *Aphos porosus*

**DOI:** 10.1038/s41598-023-40698-1

**Published:** 2023-08-25

**Authors:** Cynthia Vásquez, Iván Vera-Escalona, Antonio Brante, Francisco Silva, Eduardo Hernández-Miranda

**Affiliations:** 1 Interdisciplinary Center for Aquaculture Research (INCAR), Universidad de Concepción, Concepción, Chile; 2 Programa de Doctorado en Sistemática y Biodiversidad, Facultad de Ciencias Naturales y Oceanográficas, Universidad de Concepción, Concepción, Chile; 3 Departamento de Ecología, Facultad de Ciencias, Universidad Católica de la Santísima Concepción, Concepción, Chile; 4 Center for Research on Biodiversity and Sustainable Environments (CIBAS), Universidad Católica de la Santísima Concepción, Concepción, Chile; 5 Programa de Doctorado en Ciencias Mención en Biodiversidad y Biorecursos, Universidad Católica de la Santísima Concepción, Concepción, Chile; 6 Laboratorio de Investigación en Ecosistemas Acuáticos (LInEA), Concepción, Chile

**Keywords:** Conservation biology, Evolutionary ecology, Population dynamics, Ecology

## Abstract

Natural disturbances can modify extinction-colonization dynamics, driving changes in the genetic diversity and structure of marine populations. Along Chilean coast (36°S, 73°W), a strong hypoxic-upwelling event in 2008, and a mega earthquake-tsunami in 2010 caused mass mortality within the *Aphos porosus* population, which is a vulnerable species with low dispersal potential. We evaluated the effects of these two major disturbances on the diversity and spatial-temporal genetic structure of *Aphos porosus* in two neighboring areas that were impacted on different levels (High level: Coliumo Bay; Low level: Itata Shelf). Thirteen microsatellites (from 2008 to 2015) amplified in individuals collected from both locations were used to evaluate the effects of the two disturbances. Results showed that after the strong hypoxic-upwelling event and the mega earthquake-tsunami, *Aphos porosus* populations exhibited lower genetic diversity and less effective population sizes (*Ne* < 20), as well as asymmetries in migration and spatial-temporal genetic structure. These findings suggest a rise in extinction-recolonization dynamics in local *Aphos porosus* populations after the disturbances, which led to a loss of local genetic diversity (mainly in Coliumo Bay area impacted the most), and to greater spatial-temporal genetic structure caused by drift and gene flow. Our results suggest that continuous genetic monitoring is needed in order to assess potential risks for *Aphos porosus* in light of new natural and anthropogenic disturbances.

## Introduction

Genetic diversity is essential to the survival, adaptability, and evolution of populations^1^. Mass mortality events provoked by strong natural disturbances in marine ecosystems can modify the extinction-colonization dynamics of species, and drive changes in diversity as well as spatial-temporal genetic structures in populations, thus decreasing population sizes, modifying connectivity and, ultimately, increasing the local effects of genetic drift and gene flow^2–4^.

Marine species can be exposed to different regimes of natural disturbances, especially in the Southeast Pacific coasts where frequent hypoxic-upwelling events, and less frequent, but stronger, mega earthquakes and tsunamis tend to occur^5,6^. Several studies suggest that upwelling process, independent from hypoxic stressing conditions generated in short-time periods, can affect connectivity among local marine populations. Upwelling acts as a barrier to dispersal, resulting in growth of in genetic structure^7–9^, as observed in the *Sebastes thompsoni*^10^ fish, for instance. On the other hand, earthquakes and tsunamis can promote gene flow among populations, increasing local genetic diversity, and reducing genetic population structure. The latter was observed, for example, in the marsh plant *Carex rugulosa* after the 2011 mega earthquake-tsunami in Japan^2^. However, responses to the same disturbance can be extremely diverse among species, and will depend on the disturbance’s regime (i.e. frequency, intensity, and extent), and the life-history traits of the species affected (i.e. dispersal potential, population size, and generational time)^11^.

**Figure 1:**
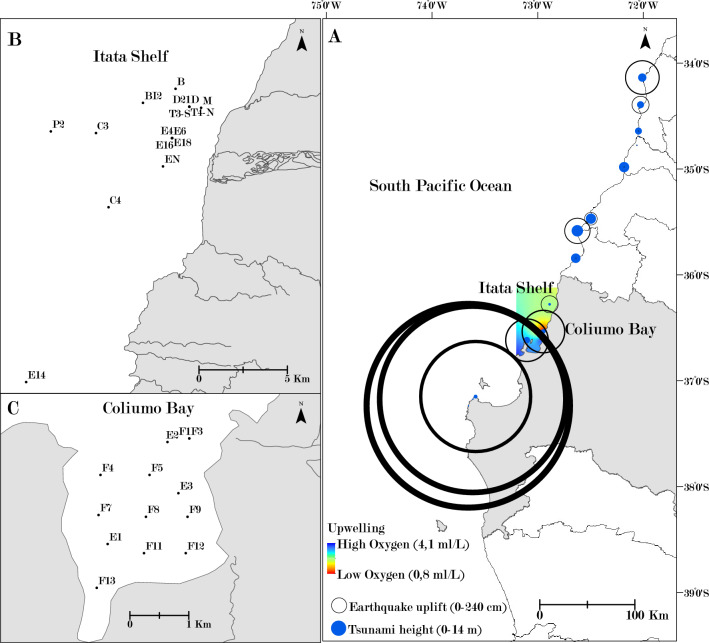
Map of the study area; (**A**). SW Pacific coast, central-southern Chile. 2008 hypoxic-upwelling event is represented with a gradient color, where high dissolved oxygen surface concentration (mL/L) is in blue, and low is in red (data obtained from Hernández-Miranda et al., 2010). The 2010 mega-earthquake uplift (cm) is represented with proportional sized black circled, and the tsunami height (m) is represented with blue proportional sized circles (data obtained from Vargas et al., 2011). (**B**). Sample stations on the Itata Shelf, and **C**. Sample stations in the Coliumo Bay, Bío Bío Region (36°S, 72°W).

Along the coast of central-southern Chile (34°–39°S), two strong natural disturbances impacted an extension of approximately 500 km of coastline. The first disturbance was a hypoxic-upwelling event (< 0.5 ml O_2_ l^−1^ in the water column), which occurred on January 3, 2008, and caused massive mortality among pelagic and benthic organisms, mainly fish species in the Coliumo Bay^12,13^. The second disturbance was a mega earthquake-tsunami (8.8 Mw, the 6th strongest earthquake since 1900), which occurred on February 27, 2010, impacting the coast along the Central-South of Chile. This event uplifted part of the coastline by more than 3 m, generating a tsunami with waves up to 14 m that devastated extensive areas of the coastal seabed and modified benthic and coastal environments, causing a massive mortality among different species in intertidal and subtidal habitats, especially in the Coliumo Bay^14–16^. Both disturbances, presented different degrees of magnitudes throughout the affected area, creating a complex patchy landscape, where the Coliumo Bay was impacted the most, and the surrounding northern area of the Itata shelf to a lesser extent. In the Itata Shelf area, the hypoxic-upwelling event decreased the dissolved oxygen on the surface, but the values remained at normoxia levels (> 2 ml O_2_ l^−1^) on the sea floor. In this area, the tsunami during the mega-earthquake was less destructive, with wave heights almost half of those in Coliumo Bay^17^ (Fig. 1).

The *Aphos porosus* toadfish (Valenciennes, 1837) was one of the species severely affected by the 2008 hypoxic-upwelling event, as well as by the 2010 mega earthquake-tsunami^18^. *Aphos* is a monotypic genus, and *Aphos porosus* is the only species of the Batrachoididae family present in Chile^19^. This benthic-demersal fish is distributed in the Southwest Pacific (3°–53°S) from Puerto Pizarro in Perú to the Strait of Magellan in Chile, and from the coast to the demersal shallow environment (0–100 m)^20–22^. *Aphos porosus* has a generation time frame of 3 years, and a breeding period that occurs in the austral spring–summer season. Females choose a protected site in intertidal pools and subtidal rocky areas, where they deposit up 167 eggs that will later be fertilized by a male. The male assists the female in spawning, and apparently secretes an adhesive substance that helps the eggs stick to the rock. Both parents care for the eggs, and the male protects the site by emitting grunts. Embryonic stages survive attached to the rocks until day 65, after which swimming larvae continue to be associated with the seabed^18,23^. The long generation time frame, and lack of pelagic behavior in larvae make these species highly vulnerable to natural and anthropogenic disturbances, given their low dispersal potential^18^.

The 2008 hypoxic-upwelling event and the 2010 mega earthquake-tsunami occurred during the *Aphos porosus* breeding season*,* in January and February, severely affecting the areas where the species lays eggs, and the larvae develop. Studies indicate that after the strong 2008 hypoxic-upwelling, the average *Aphos porosus* population density decreased drastically by 75% in the Coliumo Bay, and by 54% on the Itata Shelf, compared to 2007 before this disturbance ocurred^18^. 2 years later, after the 2010 mega earthquake-tsunami, the average *Aphos porosus* population density sharply decreased again by 83% and 64% in Coliumo Bay and Itata Shelf, respectively, compared to 2009 before this disturbance.

The main objective of this study was to evaluate the effects of the two strong natural disturbances described above (i.e., the 2008 hypoxic-upwelling event and the 2010 mega earthquake-tsunami) on the genetic diversity and structure of the *Aphos porosus* toadfish. To this end, we analyzed microsatellite markers of individuals from local populations after the two disturbances (2008–2015) in the Coliumo Bay and the Itata shelf, which are two neighboring areas with contrasting levels of impact (high for Coliumo Bay, and less for Itata shelf) due to geomorphological characteristics (Fig. 1). Given their low dispersal potential and long generation timeframe, we expected that local genetic diversity of *Aphos porosus* toadfish would decrease after the disturbances, mainly in Coliumo Bay, and that temporal genetic structure would increase between the two areas.

## Materials and methods

A total of 565 *Aphos porosus* individuals were collected from the sea floor between the years 2008 and 2015 in Coliumo Bay and Itata Shelf areas mentioned above. Benthic sampling was carried out every 3 months using a modified Agassiz trawl (1 m wide × 1 m long × 30 cm high, lined with 5 mm “knot to knot” netting). All samplings were carried out using R.V. Kay–Kay I and R.V. Kay–Kay II (Department of Oceanography, University of Concepción). The population density (individuals m^−2^) was standardized according to the number of fish collected in each tow, in relation to the swept area^12,16,18,24^. All individuals collected were genotyped using 18 microsatellite markers, previously developed by Silva et al. (2015)^25^. All experimental protocols were approved by bioethical committee of the Universidad Católica de la Santísima Concepción, Chile. Project Fondecyt Regular N° 1130868. All methods used in this study were carried out in accordance with Chilean bioethical guidelines and regulations. A physical euthanasian protocol for moribund collected fish was developed according to the “*Manual de Normas de Bioseguridad y Riesgos Asociados de FONDECYT-CONICYT*” (“Manual of Biosecurity Norms and Associated Risks by FONDECYT-CONICYT) and its references. No chemical product was used during physical euthanasia, and no laboratory experiments were carried out with collected fishes. Where applicable, all methods were reported in accordance with ARRIVE guidelines 2.0.

Microsatellite genotyping and detection of amplification errors were performed locus by locus using the automatic procedure with manual correction in Geneious v7.1^26^. Presence of null alleles and other genotyping errors were evaluated with MICRO-CHECKER v2.2.3^27^, by comparing observed genotypes to the distribution of randomized genotypes. Deviations from Hardy–Weinberg equilibrium (HWE) and Linkage disequilibrium (LD) were evaluated for each locus by area and year using a Markov chain algorithm^28^ with dememorization = 1000, batches = 100, Iterations per batch = 1,000 in GENEPOP v4.7 + ^29,30^. Due to multiple comparisons, we applied a Bonferroni correction to HWE and LD *p*-values, with a new *p*-value = 0.004.

Deviations from neutral expectations were evaluated with the Bayesian method implemented in the BayeScan v2.1^31^, using Number of pilot runs = 20, Burn-in length = 50,000, Number of outputted iterations = 100,000, and Thinning interval size = 10. To correct for multiple testing, the program computes q-values based on the posterior probability for each locus. q-values < 0.05 and α-values significantly > 0 suggest selection diversification, whereas q-values < 0.05 and α-values significantly < 0 suggest balancing or purifying selection.

Intrapopulation genetic diversity estimated over all loci by area and year were evaluated as follows: no. Alleles (Na) = number of different alleles; No. Alleles Private (Nap) = number of alleles unique to a single population; No. Alleles Common (Nac) = number of locally common alleles (frequency >  = 5%) found in 50% or fewer populations, observed heterozygosity (Ho), and unbiased expected heterozygosity (uHe). All indices were estimated in GenAlex v6.5^32^ according to Hartl and Clark (1989), as well as Peakall and Smouse (2012)^32^. Comparisons between areas, as well as before and after disturbances years (when possible) were made with the two-sample t-test, assuming unequal variances in Excel, and applying a Bonferroni correction to solve issues resulting from multiple comparisons. Effective population sizes (*N*_*e*_) for both areas were estimated with NeEstimator v2.1^33^, using Model II of the temporal method proposed by Nei and Tajima (1981)^34^, with a Jackknife confidence interval of 95% and *P*_Crit_ = 0.020, assuming a generation timeframe of tree years according to a previous study^18^. The individuals collected in 2008 from Coliumo Bay were eliminated from this analysis, so as to allow for comparisons between both areas, given that the number of individuals collected in 2008 on the Itata Shelf were insufficient for genetic analysis. Thus, individuals from 2009 were considered generation 0, and whose from 2015 were deemed generation 2.

To evaluate spatial and temporal genetic structures, a Principal Coordinate Analysis (PCoA) was carried out with GenAlex v6.5^32^, which provided a view of genetic differentiation between the areas under study (i.e., Coliumo Bay and the Itata shelf) as well as over time (2008–2015). Then, a clustering analysis was carried out with STRUCTURE v2.3^35^, so as to infer differences in population structure between the two areas and years. A mixed model was used, with *k* = 1 to *k* = 15, each with 10 replicates, burning = 50,000 steps, repeat = 1,000,000 steps, and LocPrior = 0. *k* was selected based on the highest Delta K^36^ value in the Structure Harvester^37^. Replicas of the cluster analysis were aligned by using CLUMPP v1.1.2^38^, and plotted with DISTRUCT^39^. The number of genetic clusters or populations (*k*) inferred with STRUCTURE was then contrasted with an Analysis of Molecular Variance (AMOVA) by using No. of different alleles as a distance method (F_ST_), No. of permutations = 999, considering 14 populations within 3 groups, where 1 = Coliumo Bay (with the years 2008–2011 nested), 2 = Coliumo Bay (with the years 2012–2015 nested), and 3 = Itata Shelf (with the years 2009–2015 nested) with Arlequin v3.5^40^. In addition, paired F_ST_ tests were estimated among 14 populations using No. of different alleles as a distance method (F_ST_), No. of permutations = 999, and applying Bonferroni corrections to solve issues resulting from multiple comparisons. F_IS_ was estimated for each area and year, following the method proposed by Weir and Cockerham (1984) with GENEPOP v4.7.5^29,30,41^.

Gene flow between Coliumo Bay and the Itata Shelf, as well as recent migration rates per generation (m), and posterior probabilities for individual ancestral distributions of immigrants were estimated with a Bayesian method implemented on BayesASS v3.0^42^, setting the following parameters: Generator seeds s = 100, iterations I = 10,000,000–100,000,000, burning = 1,000,000, sampling intervals n = 100, allelic frequencies a = 0.30, inbreeding coefficient f = 0.50 and migration rate m = 0.10. Convergence of the CMCMs were reviewed in Tracer v1.7^43^.

## Results

Among the 18 loci, 5 presented null alleles and were removed from the analysis, including 118 individuals presenting amplification errors. Therefore, a total of 13 loci and 447 individuals collected between 2008 and 2015 were included in the analyses (305 individuals from Coliumo Bay, and 142 from the Itata Shelf; see Table 1). HWE deviations were observed in Coliumo Bay and the Itata Shelf for all years, except in 2010 on the Itata Shelf. Coliumo Bay showed larger deviations from HWE than the Itata Shelf, which increased between 2014 and 2015 (see Supplementary Table 1). LDs were also observed in Coliumo Bay for all years, except 2011, while on the Itata Shelf they were observed only for 2009, 2014, and 2015 (see Supplementary Table 2). Furthermore, 8 of the 13 loci were shown to be under balancing or purifying selection (see Supplementary Table 3).

**Table 1:** Genetic diversity of *A. porous* populations; means and standard deviations (x ± s.d.) of all loci for each area and year, N° genotyped individuals (Ind.), N° alleles (Na), N° private alleles (Nap), N° common alleles (Nac), observed heterozygosity (Ho), heterozygosity corrected expected (uHe), Endogamy coefficient (F_IS_) over all loci, and immigration rate (m) from Itata Shelf to Coliumo Bay, and from Coliumo Bay to Itata Shelf.

Location	Year	N°	Na	Nap	Nac	Ho	uHe	F_IS_	m
		Ind	x (s.d)	x (s.d)	x (s.d)	x (s.d)	x (s.d)		x (s.d)
Coliumo Bay	2008	38	4.692 (0.511)	0.231 (0.166)	0.846 (0.249)	0.557 (0.071)	0.520 (0.047)	− 0.079	–
	2009	37	8.000 (0.920)	0.846 (0.436)	2.077 (0.473)	0.755 (0.041)	0.671 (0.031)	− 0.130	0.011 (0.010)
	2010	31	7.077 (0.930)	0.385 (0.180)	1.846 (0.390)	0.705 (0.045)	0.619 (0.031)	− 0.130	0.013 (0.013)
	2011	40	6.231 (0.735)	0.692 (0.263)	1.077 (0.309)	0.869 (0.043)	0.643 (0.031)	− 0.359	–
	2012	39	6.000 (0.892)	0.308 (0.175)	1.692 (0.328)	0.771 (0.069)	0.615 (0.047)	− 0.253	0.016 (0.011)
	2013	40	7.615 (0.665)	0.615 (0.266)	2.000 (0.376)	0.906 (0.028)	0.694 (0.027)	− 0.311	0.301 (0.084)
	2014	40	7.538 (0.748)	0.769 (0.323)	2.000 (0.424)	0.958 (0.018)	0.686 (0.019)	− 0.390	0.014 (0.014)
	2015	40	7.308 (0.711)	0.385 (0.140)	1.846 (0.355)	0.962 (0.016)	0.706 (0.026)	− 0.368	0.009 (0.009)
Itata Shelf	2009	32	7.692 (1.077)	1.100 (0.473)	1.923 (0.415)	0.945 (0.020)	0.710 (0.016)	− 0.340	0.324 (0.010)
	2010	8	4.615 (0.154)	0.200 (0.104)	1.308 (0.263)	0.922 (0.050)	0.696 (0.035)	− 0.358	0.300 (0.030)
	2012	13	4.462 (0.077)	0.100 (0.077)	0.923 (0.309)	0.851 (0.057)	0.668 (0.040)	− 0.289	0.305 (0.044)
	2013	37	6462 (0.154)	0.200 (0.104)	2.000 (0.408)	0.873 (0.049)	0.671 (0.035)	− 0.307	0.010 (0.009)
	2014	23	6231 (0.385)	0.400 (0.241)	1.538 (0.351)	0.895 (0.052)	0.678 (0.037)	− 0.321	0.320 (0.013)
	2015	29	6.923 (0.231)	0.200 (0.166)	2.154 (0.492)	0.872 (0.060)	0.679 (0.047)	− 0.297	0.320 (0.026)

Genetic diversity of *Aphos porosus* showed differences between and within areas in the years after disturbances. Coliumo Bay showed lower observed heterozygosity than the Itata Shelf in 2009 and 2010 (Table 1, see also Supplementary Table 4, and Supplementary Fig. 1). In Coliumo Bay, number of alleles (Na) was significantly lower after 2008 (“the hypoxia year”) than what was observed for 2009, 2013, 2014, and 2015 (*p* < 0.004). Similarly, observed heterozygosity (Ho) and corrected expected heterozygosity (uHe) were significantly lower after 2008 (“the hypoxia year”) and 2010 (“the mega earthquake-tsunami year”), compared to 2013, 2014, and 2015 (*p* < 0.004). On the Itata Shelf, the number alleles (Na) was significantly lower after 2010 (“the mega earthquake-tsunami year”) compared to 2009 (*p* < 0.004) (Table 1, see also Supplementary Table 4, and Supplementary Fig. 1). In both areas Endogamy coefficients (F_IS_) were negative for all the years under focus (Table 2). Harmonic means of effective population sizes between generations 0 & 2 (2009 & 2015) were *N*_*e*_ = 17.4 (95% CI 10.0–29.9) and *N*_*e*_ = 19.1 (95% CI 11.0–34.1) for Coliumo Bay and the Itata Shelf, respectively (see Supplementary Table 5).

**Table 2:** Endogamy coefficients (Fis), for each locus, year and area.

Locus	Year	Coliumo Bay							Itata Shelf					
	2008	2009	2010	2011	2012	2013	2014	2015	2009	2010	2012	2013	2014	2015
A1	0.055	− 0.071	− 0.216*	− 0.345*	− 0.422*	− 0.210	− 0.325*	− 0.341*	− 0.384*	− 0.534	− 0.258	− 0.346*	− 0.284*	− 0.306
A2	0.076	− 0.108	− 0.126	− 0.473*	0.076	0.346	− 0.288	− 0.370*	− 0.391	− 0.105	− 0.532	− 0.221	− 0.153	0.150
A4	− 0.138	− 0.226*	− 0.127	− 0.270*	− 0.182*	− 0.319*	− 0.381*	− 0.324*	− 0.357*	− 0.436	− 0.399	− 0.361*	− 0.316*	− 0.331*
A7	− 0.106	− 0.435*	− 0.247	− 0.731*	− 0.490	− 0.506*	− 0.671*	− 0.703*	− 0.292	− 0.143	− 0.143	− 0.521*	− 0.415	− 0.318
A12	− 0.052	− 0.278	− 0.265*	− 0.295	− 0.016	− 0.410	− 0.434*	− 0.409	− 0.337	− 0.333	− 0.125	− 0.252	− 0.183	− 0.446*
A8	− 0.022	0.171*	− 0.237	− 0.365	0.100	− 0.617*	− 0.459*	− 0.313*	− 0.449*	− 0.493	− 0.057	− 0.436*	− 0.428	− 0.343*
B	− 0.463*	− 0.234*	0.097*	− 0.296*	− 0.411*	− 0.255*	− 0.310*	− 0.395*	− 0.435*	− 0.383	− 0.405*	− 0.443*	− 0.434*	− 0.212
D	− 0.562*	− 0.553*	− 0.515	− 0.526*	− 0.790*	− 0.479*	− 0.457*	− 0.452*	− 0.526*	− 0.366	− 0.399	− 0.495*	− 0.467*	− 0.540*
M	0.064	0.081*	− 0.177	− 0.294	− 0.120	− 0.159	− 0.232*	− 0.187	− 0.236	− 0.155	− 0.151	− 0.139	− 0.130	− 0.132*
Z	0.038	− 0.277	0.009	− 0.298	− 0.079*	− 0.169	− 0.261	− 0.210*	− 0.364*	− 0.534	− 0.382	− 0.311	− 0.377*	− 0.335
F	0.002	− 0.310	0.054	− 0.234	− 0.268	− 0.162	− 0.412*	− 0.207	− 0.219	− 0.318	− 0.418	− 0.320*	− 0.239	− 0.239*
R	0.222	0.161*	− 0.105	− 0.119	− 0.197	− 0.105	− 0.402*	− 0.514*	− 0.081	− 0.318	− 0.151	0.032	− 0.302*	− 0.295
T	0.220*	0.133*	0.164	− 0.477*	− 0.344	− 0.459*	− 0.528*	− 0.586*	− 0.417	− 0.474	− 0.278	− 0.217	− 0.450*	− 0.275

Hardy–Weinberg deviations are indicated with an asterisk.

**Figure 2:**
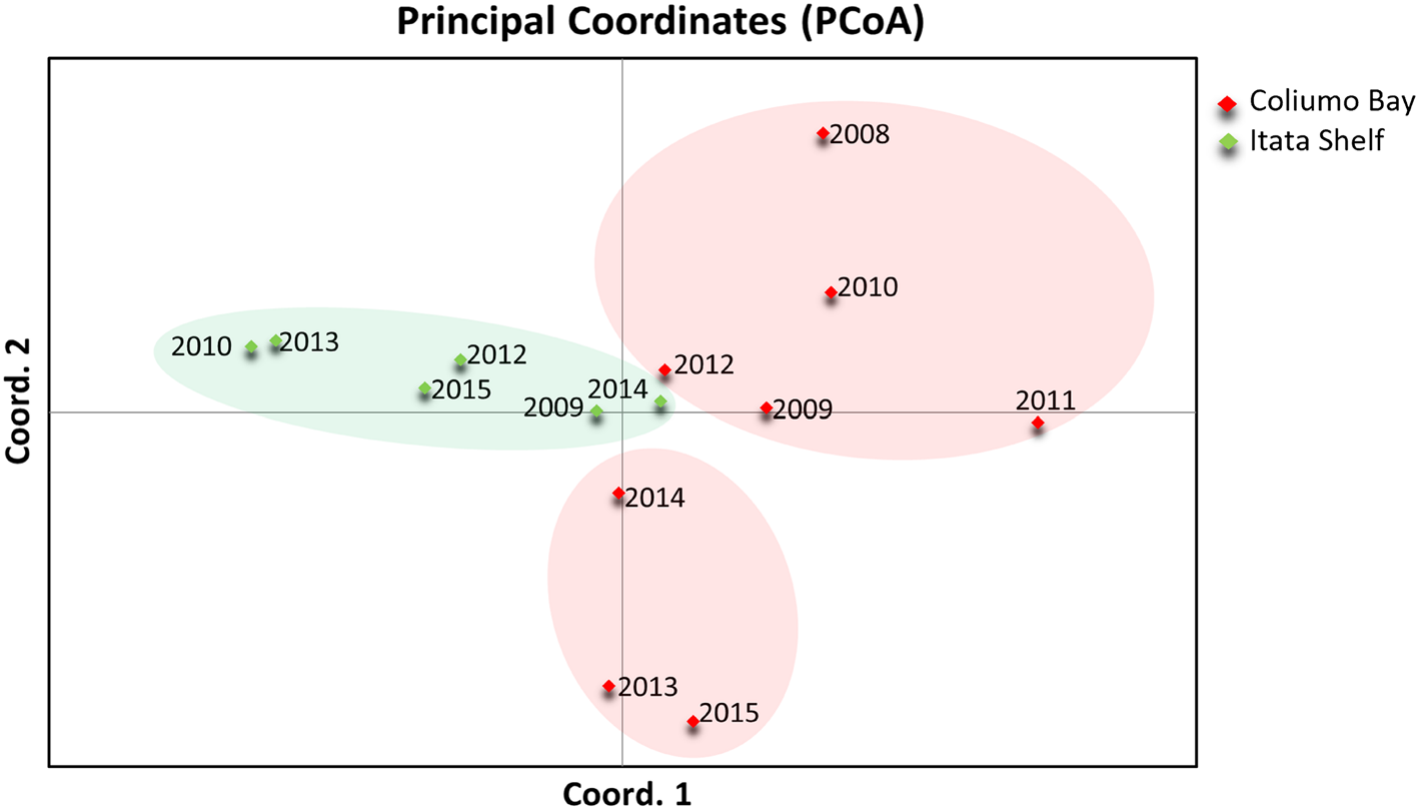
PCoA with genetic data from *Aphos porosus*, by year and area; Coliumo Bay (in red) and Itata Shelf (in green). The axis 1 and 2 explained 23.8% and 20.2% of the data variation, respectively.

**Figure 3:**
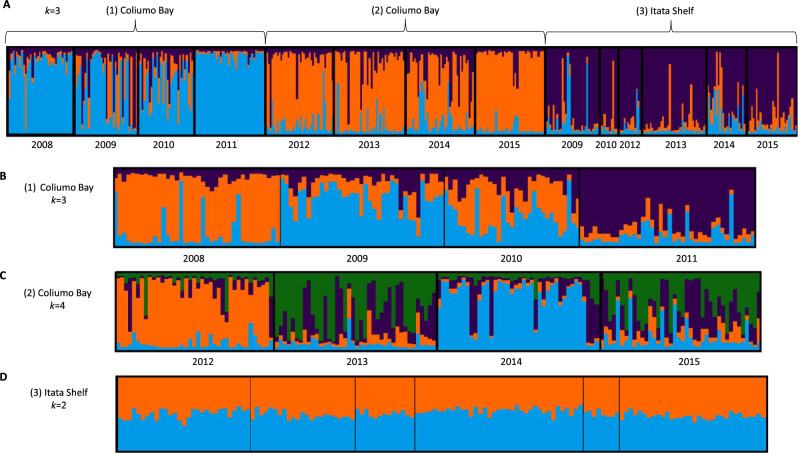
STRUCTURE Analyses of *Aphos porosus*: (**A**). Global genetic structure*, k* = 3; (1) Coliumo Bay years 2008–2011, (2) Coliumo Bay years 2012–2015, and (3) Itata Shelf years 2009–2015. (**B**), (**C**) and (**D**). Temporal STRUCTURE Analysis; (1) Coliumo Bay years 2008–2011, *k* = *3*, (2) Coliumo Bay years 2012–2015, *k* = *4* and (3) Itata Shelf 2009–2015, *k* = 2, respectively.

According to the PCoA analysis, axis 1 and 2 explained 44% of the total variance, revealing the following three clusters: (1) Coliumo Bay years 2008–2012, (2) Coliumo Bay years 2013–2015, and (3) Itata Shelf all years (Fig. 2). This clustering was similar to the results obtained from STRUCTURE, which yielded a *k* = 3 for (1) Coliumo Bay years 2008–2011, (2) Coliumo Bay years 2012–2015, and (3) Itata Shelf all years (Fig. 3A). The hierarchical analysis of STRUCTURE showed a temporal substructure in (1) Coliumo Bay, with *k* = 3 (2008, 2009–2010 and 2011) (Fig. 3B), and (2) Coliumo Bay with *k* = 4 (2012, 2013, 2014 and 2015) (Fig. 3C). The latter was in contrast to the Itata Shelf, where no temporal substructure was found (Fig. 3D).

**Figure 4:**
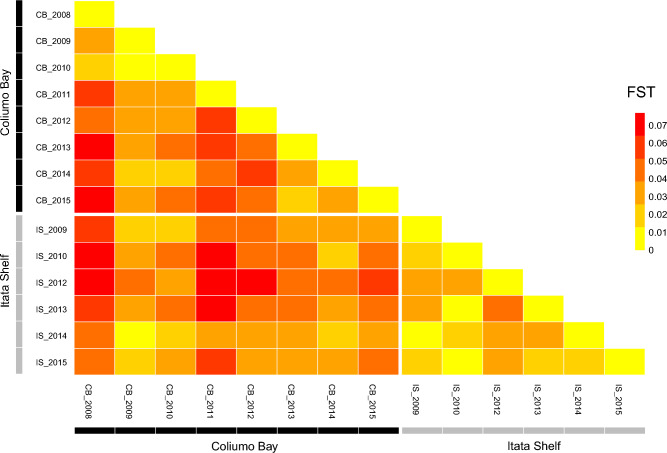
F_ST_ population pair-wise comparisons for areas and years. Distance method = No. of different alleles. All pair-wise F_ST_ comparisons were significant after Bonferroni correction (*p* < 0.004).

Results from the AMOVA analysis revealed 3 groups = 1.17%: (1) Coliumo Bay (years 2008–2011), (2) Coliumo Bay (years 2012–2015), and (3) Itata Shelf (all years included) (F_CT_ = 0.012, *p* < 0.05). Among populations within groups = 3.52%; (F_SC_ = 0.036 *p* < 0.05), and between individuals within populations = − 27.27% (F_IS_ = − 0.286, *p* > 0.05) (Table 3). All population *pair-wise* F_STs_ analyses resulted in significant values (*p* < 0.0001), but extremely small effect sizes (0.011–0.077). Genetic differentiation between Coliumo Bay and the Itata Shelf was higher in 2012 and 2013 (F_ST_ = 0.071, and F_ST_ = 0.050, respectively). The greatest difference within Coliumo Bay was observed between 2008 (“the hypoxia year”) and samples collected in 2013 and 2015 (F_ST_ = 0.076 and F_ST_ = 0.077, respectively). On the Itata Shelf, the greatest difference was observed between 2012 and 2013 (F_ST_ = 0.039), but in general, inter-annual differences were smaller compared to Coliumo Bay (Fig. 4, see also Supplementary Table 6, and Supplementary Table 7).

**Table 3:** Analysis of Molecular Variance (AMOVA); 14 subpopulations corresponding to each year by locality in 3 groups; 1 = Coliumo Bay (with the years 2008–2011 nested), 2 = Coliumo Bay (with the years 2012–2015 nested), and 3 = Itata Shelf (with the years 2009–2015).

Source of variation	d.f	Sum of squares	Variance components	Percentage of variation	Fixation indices		*P*-value
Among groups	2	58.738	0.05195 Va	1.17	F_CT_	0.012	** < 0.001**
Among populations within groups	11	140.316	0.15634 Vb	3.52	F_SC_	0.036	** < 0.001**
Among individuals within populations	433	1310.241	− 1.21241 Vc	− 27.27	F_IS_	− 0.286	> 0.05
Within individuals	447	2436.500	5.45078 Vd	122.58	F_IT_	− 0.226	> 0.05
Total	893	3945.795	4.44666				

Distance method = No. of different alleles. Fixation Indices; Differentiation Coefficient between groups (F_CT_), Differentiation Coefficient among populations withing groups (F_SC_)_,_ Inbreeding Coefficients individual relative to subpopulation (F_IS_), and Inbreeding Coefficients individual relative to total (F_IT_) significance test with 1023 permutations. In bold, significance *p*-value < 0.05.

Gene flow between Coliumo Bay and the Itata Shelf during the years after 2008 (“the hypoxia year”), was asymmetric from Coliumo Bay to the Itata Shelf. In Coliumo Bay, the rate of immigrants per generation from the Itata Shelf was nearly zero, except in 2013, where it increased to 0.3. On the Itata Shelf, the rate of immigrants per generation from Coliumo Bay was equal to 0.3, except for 2013 when gene flow decreased to 0 (Table 1).

## Discussion

Natural strong disturbances, including hypoxic-upwelling events, earthquakes, and tsunamis, have resulted in mass mortality among marine populations, increasing drift strength and affecting gene flow, causing the loss of genetic diversity as well as changes in population structure^2–4,10,44^. Understanding the relationship between demographic and genetic changes over time is of critical importance to predict resistance in at-risk populations, particularly in spatially structured populations (i.e. metapopulations) where spatial arrangements of local populations can modulate both demographic and genetic changes^45^. Considering this, we evaluated the effects of the strong 2008 hypoxic-upwelling event and the 2010 mega earthquake-tsunami on the genetic diversity and genetic structure of the *Aphos porosus* toadfish in two nearby areas, namely Coliumo Bay and the Itata Shelf, which were affected by the two disturbances to different magnitudes of impact. Results showed that genetic diversity and effective population size of *Aphos porosus* were lower after the two disturbances, mostly in the Coliumo Bay area, which was impacted the most, and exhibited greater spatial–temporal genetic structure and asymmetric migration during the years analyzed.

Biological and ecological traits of *Aphos porosus*, and particular oceanographic characteristics of the inhabiting areas can increase the probability of genetic isolation and structure, limiting the geographical distribution of this species^23,46^. The latter in combination with the impact from the disturbances, could explain the genetic diversity pattern observed. Population differentiation occurred despite the short distance between Coliumo Bay and the Itata Shelf, as suggested by the different approaches used in this study. Thus, a combined effect between ecological life history traits of the species, oceanographic conditions, and environmental stressors could explain the spatial structure pattern observed.

Firstly, *Aphos porosus* larvae have limited dispersal potential, and adult mobility is also restricted, similar to what has been observed for *Opsanus tau*, which is another Batrachoididae^47,48^. Additionally, the literature suggests temporal reproductive isolation for *Aphos porosus* populations on the latitudinal gradient, which is aligned with what has been observed in other regions of Chile^18,23^. For instance, previous studies in Chile have shown gaps of 1–2 months in the reproductive season of this species, specifically in populations in Valparaiso (32°S, 71°W), and Coliumo Bay (36°S, 72°W), which are areas separated by approximately 500 km^18,23^.

Secondly, from an oceanographic perspective, studies have suggested that upwelling^49^, which is a physical process that occurs regularly in the geographical area of south Pacific, can act as a hydrodynamic force, affecting connectivity among local populations, acting as a physical barrier to dispersal, and promoting population genetic structure, as observed in *Merluccius capensis*^9^, *Epinephelus andersoni*^7^, and *Pomacanthus maculosus*^8^ fishes in other upwelling areas, for instance. Thirdly, the 2008 hypoxic-upwelling, and the 2010 mega earthquake-tsunami caused a drastic decline in the densities of *Aphos porosus* populations (> 60%)^18^ both in the Coliumo Bay and Itata Shelf areas, and in a short time period (i.e., 2 years). This led to lower genetic diversity and a greater spatial-temporal genetic structure, similar to the effects of a bottleneck^50^. Low dispersal from the Itata Shelf to Coliumo Bay, as inferred from our estimates of gene flow, could generate temporal reproductive isolations, and genetic differentiations between both areas. Consequently, compounding factors including a generation timeframe of 3 years, spatial reproductive gaps, and oceanographic barriers could explain the population differentiation observed in these nearby areas, as well as their dissimilar responses to the same major disturbances^11,51^.

Previous studies showed that *Aphos porosus* population density in Coliumo Bay was sixty times higher than in the Itata Shelf area before the 2008 hypoxic-upwelling, which suggests that Coliumo Bay could be used as a nursey area for different fish species, including *A. porosus*^12,18^. Population density decreased in Coliumo Bay and the Itata Shelf after both disturbances. Differences between these areas before and after the disturbances could explain the asymmetries observed in migration and spatial genetic structure. Because genetic drift tends to be higher in smaller populations, the Itata Shelf could be more strongly impacted by drift, generating a differentiated population from Coliumo Bay due to rapid fixation of alleles, and the rapid loss of private alleles. Genetic structure has also been observed in the *Poricthys notatus* toadfish, the sister genus of *Aphos*, along the Pacific coast the US, as well as in *Opsanus tau* and *O. beta*, other Batrachoididae species along the Western Atlantic coast of the US^46,47,51^.

A temporal population substructure was only observed in Coliumo Bay, which may have been related to the stronger intensity of both disturbances in this area. Coliumo Bay exhibited greater genetic differentiation between 2008 (hypoxic-upwelling year) and the years 2013 and 2015 (after the mega earthquake-tsunami). Inter-annual substructures observed in Coliumo Bay may reflect the increase in drift and gene flow after disturbances, probably due to the seasonal (Summer-Autumn) recruitment of new cohorts and immigrants from neighboring localities with new alleles. Unfortunately, this hypothesis could not be corroborated, given that other surrounding areas were not genetically sampled, and the method used in this study did not include migration from unobserved populations^34^. Studies on species affected by mega earthquakes and tsunamis reveal that populations with high densities and high larval dispersal are key to maintain genetic diversity, as occurred with the snail *Batillaria attramentaria* and the sea urchin *Mesocentrotus nudus* after the 2011 Japan’s earthquake^52,53^. Other genetic studies suggest that disturbances like earthquakes and tsunamis can enable the colonization of new lineages^54,55^ from less affected areas. For example, another toadfish species from the genera *Porichthys* and *Opsanus* have been observed to raft on logs for 25 miles during the summer period, and on sponges during stormy times^56,57^. Moreover, all samples and years revealed evidence of negative F_IS_ values, which signals outbreeding that could be explained on the Itata Shelf by the high number of immigrants observed from Coliumo Bay. Heterozygosity increased by 10% in Coliumo Bay after 2011, which together with negative F_IS_ values could indicate immigration from an unsampled population, but might also signal the effects of genetic drift resulting from the disturbances. The latter is supported by the high population differentiation observed after 2011.

Drift, immigration and substructure could have also caused another pattern observed in *Aphos porosus* populations, for instance, HWE deviations and evidence of LD, which in general were higher in Coliumo Bay compared to the Itata Shelf. Deviations from HWE and LD can appear to be consequences of immigrants strongly differing genetically from local populations. This could be due to mixing and finite population abundances with higher frequencies of heterozygotes than expected, but it might also be due to substructure^58–62^.

The effects of the 2010 mega earthquake-tsunami on genetic diversity indices have been studied in other organisms, suggesting that not all species were affected similarly. For instance, the red seaweed *Agarophyton chilense* showed a population decline and a loss of 10–40% in allelic diversity, recovering 2 years post disturbance due to migration^63^. The intertidal crustaceans *Emerita analoga* and *Excirolana hirsuticauda* showed low haplotype diversity after the 2010 mega earthquake-tsunami, followed by a recovery after 3 years. As a consequence of this disturbance, *E. analoga* revealed a lower genetic differentiation and increased gene flow post disturbance, probably due to its high larval dispersal potential. Unlike *Agarophyton chilense* or *E. analoga*, *Aphos porosus* did not show recovery in genetic diversity nor density population after 5 years of the 2010 mega earthquake-tsunami. The low dispersal potential, together with the generational timeframe of 3 years, could limit their ability to recover fast in impacted areas^11,51^. *E. hirsuticauda* showed a slight increase in population differentiation, and few changes in gene flow. In this case recovery was associated with adult survival and rapid colonization, due to the strong swimming capacity of juveniles and adults^11^. Similar to what was observed for *E. hirsuticauda, Aphos porosus* showed an increase in genetic structure in Coliumo Bay after the 2010 mega earthquake-tsunami, which could probably be explained by the combined effect of increasing drift, recruitment of new cohorts from survivors, and asymmetric immigration from neighboring areas introducing new alleles.

The effects of disturbances on genetic diversity depend on both the disturbance regime and life history traits of each species, which as a whole determine the resilience capacity of the populations to resist and subsequently recovery^64^. Hence, the response of each species to the same disturbance could be extremely diverse, as shown previously^11,64^. Studies suggest that species with high population abundances, and high dispersal or mobility have higher resilience capabilities^11,51^. For *Aphos porosus*, which is a species with low dispersal potential, there are no previous studies regarding its genetic diversity and structure in its entire geographic distribution range, and there is a lack of information about the mobility capacity of adults and juveniles. Our results suggest that after the two disturbances, local populations inhabiting Coliumo Bay and the Itata Shelf decreased in genetic diversity and increased in spatial–temporal genetic structure caused by drift and gene flow.

## Conclusions

Several studies have examined how marine species can be affected by hypoxia and earthquakes separately, but not by the combined effect of both disturbances. Here, we focused on both types of disturbances, and provided evidence on how the *Aphos porosus* population responded sequentially after the two major natural disturbances considered in this study, deferring along short spatial distances with different intensities of impact. These results gain importance under a global perspective, given that natural disturbance regimes such as hypoxic-upwelling events are expected to increase due to climate change^65,66^. Results suggest that monitoring for the conservation and maintenance of genetic diversity should be an essential management priority for ensuring the future resilience and adaptive potential of marine populations worldwide^67,68^.

### Supplementary Information

Supplementary Information.

## Data Availability

The datasets generated and/or analysed during the current study are available in the following link: https://figshare.com/s/f2c8fdc28cf85a105434.
