# Supplementary material for: Natural mega disturbances drive spatial and temporal changes in diversity and genetic structure on the toadfish Aphos porosus
Source: Sci Rep. 2023 Aug 25;13:13902. doi: 10.1038/s41598-023-40698-1 (PMC10457337; doi:10.1038/s41598-023-40698-1)
Supplement: Supplementary file 1 — Supplementary Information. [file 41598_2023_40698_MOESM1_ESM.docx]

**Natural Mega Disturbances Drive Spatial and Temporal Changes in Diversity and Genetic Structure on the Toadfish *Aphos porosus***

Cynthia Vásquez^1,2^, Iván Vera-Escalona^3^, Antonio Brante^3,4,5^, Francisco Silva^5^ & Eduardo Hernández-Miranda^1,3,6^

^1^Interdisciplinary Center for Aquaculture Research (INCAR), Universidad de Concepción, Concepción, Chile.

^2^Programa de Doctorado en Sistemática y Biodiversidad, Facultad de Ciencias Naturales y Oceanográficas, Universidad de Concepción, Concepción, Chile.

^3^Departamento de Ecología, Facultad de Ciencias, Universidad Católica de la Santísima Concepción, Concepción, Chile.

^4^Center for Research on Biodiversity and Sustainable Environments (CIBAS). Universidad Católica de la Santísima Concepción, Concepción, Concepción, Chile.

^5^Programa de Doctorado en Ciencias Mención en Biodiversidad y Biorecursos, Universidad Católica de la Santísima Concepción, Concepción, Chile.

^6^Laboratorio de Investigación en Ecosistemas Acuáticos (LInEA), Concepción, Chile.

*****Corresponding author: Eduardo Hernández-Miranda, [ehernandez@ucsc.cl](mailto:ehernandez@ucsc.cl)

**Supplementary Table 1**. Hardy-Weinberg Equilibrium (HWE) deviations for all loci, as well as each area and year. Probability-test estimation of exact p-values with the Markov chain method. Total number loci per year and area deviated from HWE; significance level after Bonferroni correction p<0.004*.

| Locality | Coliumo Bay | | | | | | | | Itata Shelf | | | | | |
| --- | --- | --- | --- | --- | --- | --- | --- | --- | --- | --- | --- | --- | --- | --- |
| Loci | 2008 | 2009 | 2010 | 2011 | 2012 | 2013 | 2014 | 2015 | 2009 | 2010 | 2012 | 2013 | 2014 | 2015 |
| A1 | 0.606 | 0.018 | 0.001* | 0.001* | 0.000* | 0.005 | 0.001* | 0.003* | 0.000* | 0.248 | 0.687 | 0.000* | 0.000* | 0.052 |
| A2 | 0.538 | 0.887 | 0.005 | 0.000* | 0.548 | 0.155 | 0.007 | 0.000* | 0.007 | 1.000 | 0.035 | 0.370 | 1.000 | 0.433 |
| A4 | 0.535 | 0.001* | 0.175 | 0.001* | 0.001* | 0.000* | 0.000* | 0.000* | 0.000* | 0.094 | 0.040 | 0.003* | 0.002* | 0.000* |
| A7 | 1.000 | 0.000* | 0.616 | 0.000* | 0.006 | 0.000* | 0.000* | 0.000* | 0.372 | 0.045 | 1.000 | 0.000* | 0.005 | 0.821 |
| A12 | 1.000 | 0.035 | 0.001* | 0.303 | 0.623 | 0.061 | 0.000* | 0.008 | 0.022 | 1.000 | 0.067 | 0.098 | 0.419 | 0.001* |
| A8 | 0.032 | 0.001* | 0.481 | 0.019 | 0.647 | 0.000* | 0.000* | 0.000* | 0.000* | 0.238 | 0.314 | 0.000* | 0.013 | 0.000* |
| B | 0.000* | 0.001* | 0.000* | 0.003* | 0.001* | 0.003* | 0.000* | 0.000* | 0.000* | 0.645 | 0.003* | 0.000* | 0.000* | 0.154 |
| D | 0.000* | 0.000* | 0.000* | 0.000* | 0.000* | 0.000* | 0.000* | 0.000* | 0.000* | 0.162 | 0.024 | 0.000* | 0.000* | 0.000* |
| M | 0.317 | 0.000* | 0.048 | 0.000* | 0.125 | 0.098 | 0.002* | 0.012 | 0.006 | 0.061 | 0.491 | 0.096 | 0.636 | 0.000* |
| Z | 0.051 | 0.114 | 0.488 | 0.018 | 0.000* | 0.081 | 0.131 | 0.000* | 0.001* | 0.125 | 0.200 | 0.005 | 0.000* | 0.655 |
| F | 0.050 | 0.006 | 0.038 | 0.264 | 0.180 | 0.033 | 0.000* | 0.037 | 0.030 | 0.141 | 0.119 | 0.001* | 0.018 | 0.296 |
| R | 0.189 | 0.000* | 0.007 | 0.566 | 0.301 | 0.978 | 0.000* | 0.000* | 0.291 | 0.078 | 0.739 | 0.082 | 0.000* | 0.000* |
| T | 0.002* | 0.000* | 0.283 | 0.000* | 0.109 | 0.000* | 0.001* | 0.000* | 0.007 | 0.305 | 1.000 | 0.007 | 0.000* | 0.485 |
| Total | 3 | 8 | 4 | 8 | 5 | 6 | 11 | 10 | 7 | 0 | 1 | 7 | 7 | 6 |

| **Supplementary Table 2.** Linkage disequilibrium (LD): Numbers of loci pairs with significance levels after Bonferroni correction (p<0.004) for each locus, area, and year. Likelihood ratio statistic (G-test) using a Markov chain algorithm.   \|  \| Coliumo Bay \| \| \| \| \| \| \| \| \| Itata Shelf \| \| \| \| \| \| \| \| --- \| --- \| --- \| --- \| --- \| --- \| --- \| --- \| --- \| --- \| --- \| --- \| --- \| --- \| --- \| --- \| --- \| \| Loci \| 2008 \| 2009 \| 2010 \| 2011 \| 2012 \| 2013 \| 2014 \| 2015 \| Total \| 2009 \| 2010 \| 2012 \| 2013 \| 2014 \| 2015 \| Total \| \| A1 \| 0 \| 0 \| 0 \| 0 \| 0 \| 0 \| 1 \| 0 \| 1 \| 0 \| 0 \| 0 \| 0 \| 0 \| 0 \| 0 \| \| A2 \| 0 \| 0 \| 1 \| 0 \| 0 \| 1 \| 0 \| 0 \| 2 \| 0 \| 0 \| 0 \| 0 \| 0 \| 0 \| 0 \| \| A4 \| 0 \| 0 \| 0 \| 0 \| 0 \| 0 \| 0 \| 0 \| 0 \| 0 \| 0 \| 0 \| 0 \| 0 \| 0 \| 0 \| \| A7 \| 0 \| 0 \| 1 \| 0 \| 0 \| 0 \| 0 \| 0 \| 1 \| 0 \| 0 \| 0 \| 0 \| 0 \| 0 \| 0 \| \| A12 \| 0 \| 0 \| 0 \| 0 \| 0 \| 0 \| 1 \| 0 \| 1 \| 0 \| 0 \| 0 \| 0 \| 0 \| 0 \| 0 \| \| A8 \| 0 \| 1 \| 0 \| 0 \| 1 \| 0 \| 0 \| 1 \| 3 \| 0 \| 0 \| 0 \| 0 \| 0 \| 1 \| 1 \| \| B \| 0 \| 0 \| 0 \| 0 \| 0 \| 0 \| 1 \| 0 \| 1 \| 0 \| 0 \| 0 \| 0 \| 0 \| 0 \| 0 \| \| D \| 0 \| 0 \| 0 \| 0 \| 0 \| 0 \| 0 \| 0 \| 0 \| 0 \| 0 \| 0 \| 0 \| 1 \| 0 \| 1 \| \| M \| 0 \| 0 \| 0 \| 0 \| 0 \| 0 \| 0 \| 0 \| 0 \| 0 \| 0 \| 0 \| 0 \| 0 \| 0 \| 0 \| \| Z \| 1 \| 0 \| 0 \| 0 \| 0 \| 1 \| 1 \| 0 \| 3 \| 0 \| 0 \| 0 \| 0 \| 0 \| 0 \| 0 \| \| F \| 1 \| 0 \| 0 \| 0 \| 0 \| 0 \| 0 \| 0 \| 1 \| 1 \| 0 \| 0 \| 0 \| 1 \| 0 \| 2 \| \| R \| 0 \| 0 \| 0 \| 0 \| 0 \| 0 \| 0 \| 0 \| 0 \| 0 \| 0 \| 0 \| 0 \| 0 \| 0 \| 0 \| \| T \| 0 \| 1 \| 0 \| 0 \| 1 \| 0 \| 0 \| 1 \| 3 \| 1 \| 0 \| 0 \| 0 \| 0 \| 1 \| 2 \| \| Total \| 2 \| 2 \| 2 \| 0 \| 2 \| 2 \| 4 \| 2 \| 16 \| 2 \| 0 \| 0 \| 0 \| 2 \| 2 \| 6 \| | | | | | | | | | | | | | | | |  |  |
| --- | --- | --- | --- | --- | --- | --- | --- | --- | --- | --- | --- | --- | --- | --- | --- | --- | --- | --- | --- | --- | --- | --- | --- | --- | --- | --- | --- | --- | --- | --- | --- | --- | --- | --- | --- | --- | --- | --- | --- | --- | --- | --- | --- | --- | --- | --- | --- | --- | --- | --- | --- | --- | --- | --- | --- | --- | --- | --- | --- | --- | --- | --- | --- | --- | --- | --- | --- | --- | --- | --- | --- | --- | --- | --- | --- | --- | --- | --- | --- | --- | --- | --- | --- | --- | --- | --- | --- | --- | --- | --- | --- | --- | --- | --- | --- | --- | --- | --- | --- | --- | --- | --- | --- | --- | --- | --- | --- | --- | --- | --- | --- | --- | --- | --- | --- | --- | --- | --- | --- | --- | --- | --- | --- | --- | --- | --- | --- | --- | --- | --- | --- | --- | --- | --- | --- | --- | --- | --- | --- | --- | --- | --- | --- | --- | --- | --- | --- | --- | --- | --- | --- | --- | --- | --- | --- | --- | --- | --- | --- | --- | --- | --- | --- | --- | --- | --- | --- | --- | --- | --- | --- | --- | --- | --- | --- | --- | --- | --- | --- | --- | --- | --- | --- | --- | --- | --- | --- | --- | --- | --- | --- | --- | --- | --- | --- | --- | --- | --- | --- | --- | --- | --- | --- | --- | --- | --- | --- | --- | --- | --- | --- | --- | --- | --- | --- | --- | --- | --- | --- | --- | --- | --- | --- | --- | --- | --- | --- | --- | --- | --- | --- | --- | --- | --- | --- | --- | --- | --- | --- | --- | --- | --- | --- | --- | --- | --- | --- | --- | --- | --- | --- | --- | --- | --- | --- | --- | --- | --- | --- | --- | --- | --- | --- | --- | --- | --- | --- | --- | --- | --- | --- | --- | --- | --- | --- | --- | --- | --- | --- | --- | --- | --- | --- | --- | --- | --- | --- | --- | --- |
|  |  |  |  |  |  |  |  |  |  |  |  |  |  |  |  |  |  |
|  |  |  |  |  |  |  |  |  |  |  |  |  |  |  |  |  |  |

**Supplementary Table 3.** Deviations of neutrality for each locus. Bayesian Method, where Prob = the posterior probability for the model including selection. Log_10_(PO) = the logarithm of posterior odds to base 10 for the model including selection, qval = posterior probability for each locus corrected, Alfa = coefficient indicating the strength and direction of selection. In bold q-values < 0.05 and α-values significantly > 0 suggests selection diversification, whereas q-values < 0.05 and α-values significantly < 0 suggest balancing or purifying selection.

| Loci | Prob | Log_10_(PO) | qval | Alpha |
| --- | --- | --- | --- | --- |
| A1 | 1.000 | 1000.000 | **0.000** | **-1.376** |
| A2 | 0.024 | -1.617 | 0.374 | 0.001 |
| A4 | 1.000 | 1000.000 | **0.000** | **-2.410** |
| A7 | 0.042 | -1.358 | 0.108 | 0.011 |
| A12 | 1.000 | 1000.000 | **0.000** | **-1.512** |
| A8 | 0.026 | -1.567 | 0.265 | 0.000 |
| B | 1.000 | 1000.000 | **0.000** | **-1.842** |
| D | 1.000 | 1000.000 | **0.000** | **-2.045** |
| M | 1.000 | 1000.000 | **0.000** | **-1.776** |
| Z | 0.986 | 1.841 | **0.002** | **-0.740** |
| F | 1.000 | 1000.000 | **0.000** | **-1.430** |
| R | 0.032 | -1.483 | 0.194 | -0.003 |
| T | 0.025 | -1.584 | 0.324 | -0.005 |

**Supplementary Table 4**. Two-sample t-test assuming unequal variances to N° alleles (Na), N° private alleles (Nap), N° common alleles (Nac), observed heterozygosity (Ho) and corrected expected heterozygosity (uHe). p-value between years within each area and between areas for each year. Significance level after Bonferroni correction p<0.004*.

| Locality | Years | Na | Nap | Nac | Ho | uHe |
| --- | --- | --- | --- | --- | --- | --- |
| Coliumo Bay | 2008-2009 | 0.003* | 0.104 | 0.017 | 0.013 | 0.007 |
|  | 2008-2010 | 0.018 | 0.268 | 0.021 | 0.047 | 0.048 |
|  | 2008-2011 | 0.050 | 0.077 | 0.283 | 0.001* | 0.021 |
|  | 2008-2012 | 0.109 | 0.376 | 0.026 | 0.020 | 0.085 |
|  | 2008-2013 | 0.001* | 0.076 | 0.009 | 0.000* | 0.002* |
|  | 2008-2014 | 0.002* | 0.078 | 0.015 | 0.000* | 0.002* |
|  | 2008-2015 | 0.003* | 0.116 | 0.016 | 0.000* | 0.012 |
|  | 2009-2010 | 0.244 | 0.171 | 0.355 | 0.209 | 0.126 |
|  | 2009-2011 | 0.073 | 0.383 | 0.046 | 0.033 | 0.266 |
|  | 2009-2012 | 0.066 | 0.134 | 0.256 | 0.420 | 0.168 |
|  | 2009-2013 | 0.369 | 0.328 | 0.320 | 0.003* | 0.287 |
|  | 2009-2014 | 0.350 | 0.444 | 0.452 | 0.000* | 0.336 |
|  | 2009-2015 | 0.279 | 0.166 | 0.350 | 0.000* | 0.195 |
|  | 2010-2011 | 0.241 | 0.173 | 0.068 | 0.007* | 0.299 |
|  | 2010-2012 | 0.206 | 0.381 | 0.383 | 0.215 | 0.471 |
|  | 2010-2013 | 0.321 | 0.241 | 0.389 | 0.001* | 0.042 |
|  | 2010-2014 | 0.351 | 0.156 | 0.396 | 0.000* | 0.042 |
|  | 2010-2015 | 0.423 | 0.500 | 0.500 | 0.000* | 0.022 |
| Itata Shelf | 2009-2010 | 0.000* | 0.040 | 0.112 | 0.338 | 0.365 |
|  | 2009-2012 | 0.000* | 0.029 | 0.033 | 0.070 | 0.171 |
|  | 2009-2013 | 0.097 | 0.040 | 0.448 | 0.098 | 0.165 |
|  | 2009-2014 | 0.032 | 0.104 | 0.243 | 0.195 | 0.225 |
|  | 2009 -2015 | 0.200 | 0.056 | 0.362 | 0.133 | 0.270 |
|  | 2010-2012 | 0.400 | 0.279 | 0.176 | 0.179 | 0.299 |
|  | 2010-2013 | 0.018 | 0.500 | 0.084 | 0.248 | 0.311 |
|  | 2010-2014 | 0.009 | 0.196 | 0.302 | 0.359 | 0.365 |
|  | 2010-2015 | 0.005 | 0.350 | 0.073 | 0.266 | 0.386 |
| Coliumo & Itata | 2009 | 0.389 | 0.362 | 0.405 | 0.000* | 0.137 |
|  | 2010 | 0.013 | 0.141 | 0.132 | 0.002* | 0.058 |
|  | 2012 | 0.073 | 0.122 | 0.050 | 0.193 | 0.201 |
|  | 2013 | 0.127 | 0.063 | 0.500 | 0.285 | 0.307 |
|  | 2014 | 0.081 | 0.175 | 0.205 | 0.138 | 0.426 |
|  | 2015 | 0.352 | 0.243 | 0.309 | 0.085 | 0.310 |

| **Supplementary Table 5.** Effective population size (*Ne*) for each area. Temporal method proposed by Nei and Tajima (1981), with a Jackknife confidence interval of 95%. Generations 0.0 (2009) & 2.0 (2015). | | | | | | | | | |
| --- | --- | --- | --- | --- | --- | --- | --- | --- | --- |
|  | Coliumo Bay | | | | Itata Shelf | | | |  |
| Lowest Allele Frequency Used | 0.050 | 0.020 | 0.010 | 0+ | 0.050 | 0.020 | 0.010 | 0+ |  |
| Independent Alleles | 48 | 70 | 99 | 123 | 49 | 76 | 98 | 115 |  |
| *Ne* | 14.7 | 17.4 | 24.0 | 29.1 | 20.9 | 19.1 | 23.9 | 27.4 |  |
| Jackknife on Loci Low | 8.0 | 10.0 | 13.6 | 17.3 | 9.5 | 11.0 | 13.9 | 16.6 |  |
| Jackknife on Loci High | 26.1 | 29.9 | 43.4 | 51.3 | 50.2 | 34.1 | 43.3 | 48.2 |  |

**Supplementary Table 6**. Pair-wise populations F_ST_ values per area and year. Distance method = No. of different alleles.

|  |  | Coliumo Bay | | | | | | | | Itata Shelf | | | | | |
| --- | --- | --- | --- | --- | --- | --- | --- | --- | --- | --- | --- | --- | --- | --- | --- |
|  | Year | 2008 | 2009 | 2010 | 2011 | 2012 | 2013 | 2014 | 2015 | 2009 | 2010 | 2012 | 2013 | 2014 | 2015 |
| Coliumo Bay | 2008 | 0.000 |  |  |  |  |  |  |  |  |  |  |  |  |  |
|  | 2009 | 0.037 | 0.000 |  |  |  |  |  |  |  |  |  |  |  |  |
|  | 2010 | 0.013 | 0.013 | 0.000 |  |  |  |  |  |  |  |  |  |  |  |
|  | 2011 | 0.061 | 0.031 | 0.028 | 0.000 |  |  |  |  |  |  |  |  |  |  |
|  | 2012 | 0.047 | 0.033 | 0.034 | 0.064 | 0.000 |  |  |  |  |  |  |  |  |  |
|  | 2013 | 0.076 | 0.038 | 0.044 | 0.054 | 0.043 | 0.000 |  |  |  |  |  |  |  |  |
|  | 2014 | 0.057 | 0.019 | 0.026 | 0.043 | 0.055 | 0.038 | 0.000 |  |  |  |  |  |  |  |
|  | 2015 | 0.077 | 0.029 | 0.040 | 0.057 | 0.048 | 0.015 | 0.027 | 0.000 |  |  |  |  |  |  |
| Itata Shelf | 2009 | 0.051 | 0.019 | 0.018 | 0.043 | 0.042 | 0.033 | 0.030 | 0.037 | 0.000 |  |  |  |  |  |
|  | 2010 | 0.067 | 0.034 | 0.041 | 0.068 | 0.045 | 0.039 | 0.023 | 0.048 | 0.025 | 0.000 |  |  |  |  |
|  | 2012 | 0.067 | 0.040 | 0.034 | 0.067 | 0.071 | 0.041 | 0.042 | 0.057 | 0.026 | 0.030 | 0.000 |  |  |  |
|  | 2013 | 0.060 | 0.037 | 0.041 | 0.076 | 0.042 | 0.050 | 0.034 | 0.050 | 0.027 | 0.004 | 0.039 | 0.000 |  |  |
|  | 2014 | 0.043 | 0.013 | 0.015 | 0.035 | 0.036 | 0.032 | 0.022 | 0.032 | 0.011 | 0.020 | 0.031 | 0.034 | 0.000 |  |
|  | 2015 | 0.050 | 0.025 | 0.032 | 0.058 | 0.035 | 0.032 | 0.028 | 0.041 | 0.022 | 0.011 | 0.035 | 0.015 | 0.017 | 0.000 |

**Supplementary Table 7.** Pair-wise population F_ST_ p-values +/- standard deviations per area and year. Distance method = No. of different alleles. Significance level after Bonferroni correction p<0.004.

|  |  | Coliumo Bay | | | | | | | | Itata Shelf | | | | | |
| --- | --- | --- | --- | --- | --- | --- | --- | --- | --- | --- | --- | --- | --- | --- | --- |
|  | Years | 2008 | 2009 | 2010 | 2011 | 2012 | 2013 | 2014 | 2015 | 2009 | 2010 | 2012 | 2013 | 2014 | 2015 |
| Coliumo Bay | 2008 | * |  |  |  |  |  |  |  |  |  |  |  |  |  |
|  | 2009 | 0.00000+-0.0000 | * |  |  |  |  |  |  |  |  |  |  |  |  |
|  | 2010 | 0.00098+-0.0010 | 0.00000+-0.0000 | * |  |  |  |  |  |  |  |  |  |  |  |
|  | 2011 | 0.00000+-0.0000 | 0.00000+-0.0000 | 0.00000+-0.0000 | * |  |  |  |  |  |  |  |  |  |  |
|  | 2012 | 0.00000+-0.0000 | 0.00000+-0.0000 | 0.00000+-0.0000 | 0.00000+-0.0000 | * |  |  |  |  |  |  |  |  |  |
|  | 2013 | 0.00000+-0.0000 | 0.00000+-0.0000 | 0.00000+-0.0000 | 0.00000+-0.0000 | 0.00000+-0.0000 | * |  |  |  |  |  |  |  |  |
|  | 2014 | 0.00000+-0.0000 | 0.00000+-0.0000 | 0.00000+-0.0000 | 0.00000+-0.0000 | 0.00000+-0.0000 | 0.00000+-0.0000 | * |  |  |  |  |  |  |  |
|  | 2015 | 0.00000+-0.0000 | 0.00000+-0.0000 | 0.00000+-0.0000 | 0.00000+-0.0000 | 0.00000+-0.0000 | 0.00000+-0.0000 | 0.00000+-0.0000 | * |  |  |  |  |  |  |
| Itata Shelf | 2009 | 0.00000+-0.0000 | 0.00000+-0.0000 | 0.00000+-0.0000 | 0.00000+-0.0000 | 0.00000+-0.0000 | 0.00000+-0.0000 | 0.00000+-0.0000 | 0.00000+-0.0000 | * |  |  |  |  |  |
|  | 2010 | 0.00000+-0.0000 | 0.00000+-0.0000 | 0.00000+-0.0000 | 0.00000+-0.0000 | 0.00000+-0.0000 | 0.00000+-0.0000 | 0.00000+-0.0000 | 0.00000+-0.0000 | 0.00000+-0.0000 | * |  |  |  |  |
|  | 2012 | 0.00000+-0.0000 | 0.00000+-0.0000 | 0.00000+-0.0000 | 0.00000+-0.0000 | 0.00000+-0.0000 | 0.00000+-0.0000 | 0.00000+-0.0000 | 0.00000+-0.0000 | 0.00000+-0.0000 | 0.00000+-0.0000 | * |  |  |  |
|  | 2013 | 0.00000+-0.0000 | 0.00000+-0.0000 | 0.00000+-0.0000 | 0.00000+-0.0000 | 0.00000+-0.0000 | 0.00000+-0.0000 | 0.00000+-0.0000 | 0.00000+-0.0000 | 0.00000+-0.0000 | 0.00781+-0.0031 | 0.00000+-0.0000 | * |  |  |
|  | 2014 | 0.00000+-0.0000 | 0.00000+-0.0000 | 0.00000+-0.0000 | 0.00000+-0.0000 | 0.00000+-0.0000 | 0.00000+-0.0000 | 0.00000+-0.0000 | 0.00000+-0.0000 | 0.00000+-0.0000 | 0.00000+-0.0000 | 0.00000+-0.0000 | 0.00000+-0.0000 | * |  |
|  | 2015 | 0.00000+-0.0000 | 0.00000+-0.0000 | 0.00000+-0.0000 | 0.00000+-0.0000 | 0.00000+-0.0000 | 0.00000+-0.0000 | 0.00000+-0.0000 | 0.00000+-0.0000 | 0.00000+-0.0000 | 0.00000+-0.0000 | 0.00000+-0.0000 | 0.00000+-0.0000 | 0.00000+-0.0000 | * |

**A**

**B**

**D**

**C**

*2008<2009, 2013-2015


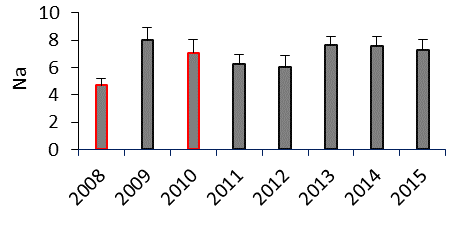


*

**F**

**E**

**H**

**G**

*2008-2010<2011, 2013-2015

**Supplementary Figure 1.** Total density (N° Ind./500 m^2^) of *Aphos porosus* for each year in Coliumo Bay (A) and the Itata Shelf (B). See Hernández-Miranda *et al.* (2012). Means and deviations for numbers of different alleles (Na), number of alleles common (Nac), numbers of alleles unique to a single population (Nap), observed heterozygosity (Ho), unbiased expected heterozygosity (uHe) for each year in Coliumo Bay (C, E, and G) and the Itata Shelf areas (D, F, and H). The red edge in bars indicates the 2008 hypoxic-upwelling event, and the 2010 mega earthquake-tsunami. T-test significant differences between years *p<0.004 after Bonferroni correction.
